# Developing Fluopyram as a Tool to Combat Beech Leaf Disease in Managed Landscapes and Nurseries

**DOI:** 10.2478/jofnem-2025-0042

**Published:** 2025-10-05

**Authors:** Matthew A. Borden, Paulo Vieira, Caitlin Littlejohn, Jacob Zack, Michael Sherwood, Amber Stiller, Kelby Fite, Andrew L. Loyd

**Affiliations:** Bartlett Tree Research Laboratories, Charlotte, NC; Mycology and Nematology Genetic Diversity and Biology Laboratory, United States Department of Agriculture—Agricultural Research Service, Beltsville, MD; Botanical Research Institute of Texas, Fort Worth, TX

**Keywords:** beech, beech leaf disease, BLD, broadform, *Fagus grandifolia*, *Fagus sylvatica*, fluopyram, foliar nematode, invasive pest, IPM, *Litylenchus crenatae mccannii*, management, nematicide

## Abstract

Beech leaf disease (BLD), caused by the anguinid nematode *Litylenchus crenatae mccannii* (Lcm), has recently emerged as a severe threat to beech trees (*Fagus* spp.) in eastern North America. In response, the scientific community has accelerated research on this invasive plant-parasitic nematode (PPN). Advances in BLD pathophysiology can be useful for developing management strategies. However, characteristics of both the pest and host trees make BLD uniquely challenging to manage, leaving arborists, nursery managers, and plant health care specialists with limited treatment options. The first treatment demonstrated to directly affect Lcm and suppress BLD was a late-summer foliar application program using fluopyram. These three sequential field trials explore several variables that must be determined when developing a novel management program: site appropriateness, product dosage, and the timing of foliar applications. The results support the efficacy of fluopyram-based programs in suppressing BLD but emphasize the importance of site conditions, noting that dense beech forests are unlikely to benefit from this treatment. The results also show that significantly reduced product doses can yield excellent control. Finally, the findings suggest that initiating the foliar application program earlier in the summer could be equally or more effective than beginning in late summer. This information will guide the implementation of novel management programs tailored to address the growing threat of BLD.

## Introduction

In the years since beech leaf disease (BLD) was discovered in Lake County, OH, in 2012, concern for the future of beech (*Fagus* spp.) has intensified, as the disease has rapidly spread throughout much of the Northeastern and Mid-Atlantic regions of the United States (Volk and Martin, 2023) and into adjacent regions in ON, Canada (Ewing et al., 2019; Fitza et al., 2024). Caused by the nematode *Litylenchus crenatae mccannii* (Lcm, family Anguinidae; Carta et al., 2020), BLD remains a relatively new and emerging pest of American beech, *Fagus grandifolia* Ehrh. Nevertheless, BLD has already been recognized among the most concerning current or potential high-impact non-indigenous threats to North American forests (Kantor et al., 2022). In forest settings, BLD is not only a driving force of significant beech mortality (Shepherd et al., 2025) but also exacerbates the ongoing and expanding impacts of the beech bark disease complex, caused by *Cryptococcus fagisuga* Lindinger and *Nectria coccinea* var. *faginata* (Pers.) Fr. (D’Amato et al., 2023; Loehle et al., 2024). Additionally, BLD increasingly affects not only American beech but also European beech (Colbert-Pitts et al., 2025) grown in nurseries, landscapes, and arboreta. Some nursery growers have voluntarily halted beech production and sales to limit further spread of the nematode—often at significant financial loss given the age and value of affected trees (nurserymen Bill Hendricks and John Verderber, pers. comm.).

Despite mounting economic concerns for high-value ornamental beech and environmental concerns for beech-dominated forest ecosystems, the development of management options for both ornamental industry stakeholders and forest preservation has been slow. Several factors are thought to have contributed to this delay. The novelty of the problem, including difficulty in identifying and defining Lcm as the primary causal agent and uncertainly surrounding its provenance status, has impeded the publicity and research momentum typically seen with other high-impact non-indigenous tree pests (Campbell, 2022; Popkin, 2023).

The lack of initial chemical management tools is largely due to the unusual nature of both the pest and the infection court. Typically, emergent invertebrate pests share traits with previously researched pests, and thus, a management program with existing tools can be adapted and modified to fit the need. In contrast, BLD is caused by a destructive foliar nematode, a pest type for which very few effective, registered management products were previously in use. While superficially comparable, other foliar nematode pests of ornamentals, primarily of the genus *Aphelenchoides* Fischer, 1894 (family Aphelenchoididae), are typically managed through sanitation and roguing of herbaceous host plants. However, Lcm affects both young and mature beech trees with aesthetic and ecological value that cannot be easily replaced. Critical knowledge gaps in Lcm pathophysiology, such as a limited understanding of the Lcm life cycle, the absence of a known primary vector that could be intercepted, and the unusual challenge of nematode activity within developing buds (i.e., a physically protected and difficult-to-reach target site), further prevented a rapid adaptation of existing management tools for this pest. The concurrent affliction of BLD on natural forests, ornamental production, and designed landscapes further complicates management efforts as each context requires development and testing of situationally appropriate and registered tools.

Recently, much progress has been made in understanding the causal agent of BLD and its deleterious effects on beech trees and the ecosystems they inhabit. Several recent studies have significantly advanced our knowledge of BLD pathophysiology. For example, Reed et al. (2020) characterized the population dynamics of Lcm, showing that activity in the foliage is unusually limited to the late season, peaking around September. Goraya et al. (2024) demonstrated the potential for local Lcm inoculum movement via abiotic factors, primarily wind and humidity. Vieira et al. (2023) disclosed the dramatic Lcm-induced disruptions to cell development and leaf morphology within the bud, revealing that higher Lcm numbers are associated with increasingly disorganized, hypertrophied, and abnormal cell layers that disrupt leaf primordium development and result in the symptoms observed at leaf expansion. Recent physiological studies have further elucidated the relationship between BLD symptoms and long-term decline of affected beech, including reduced photosynthetic rate, diminishing carbon assimilation capacity, and altered resource allocation, among other measurable physiological consequences (McIntire, 2023; Fletcher et al., 2024). These findings suggest that BLD is a chronic, resource-depleting disease, and may help explain variability in observed severity. Additionally, Bashian-Victoroff et al. (2023) brought attention to the belowground community dynamics of affected beech, finding evidence that increasing BLD severity correlates with a reduction in ectomycorrhizal colonization of root tips, likely a result of limited photosynthetic capacity and carbohydrate contribution. These recent studies inform management research by identifying a key priority for timing and target, that is: take action to prevent the chronic physiological harm caused by BLD, which is initiated within the bud stage.

To date, two distinct approaches have demonstrated direct activity against the causal agent, Lcm, and subsequently reduce BLD symptoms. The first is a foliar application program using the nematicide fluopyram (Loyd et al., 2024), and the second is a tree injection method using the anthelmintic thiabendazole (Loyd et al., 2025). In addition to the direct management strategies for Lcm highlighted above, phosphonate-based products (i.e., potassium phosphite) are also currently incorporated in beech management programs. While phosphonate products applied as a soil drench have not resulted in significant short-term reduction of Lcm populations in leaf or bud tissues (Loyd et al., 2024), long-term use has been associated with improved health in young beech trees and slower disease progression (Herms et al., 2023).

In this study, we explored the use of foliar applications of the nematicide fluopyram against Lcm. Fluopyram, belonging to the pyridinyl-ethylbenzamides chemical class, functions as both a FRAC Group 7 fungicide and a Group N-3 nematicide via inhibition of mitochondrial complex II electron transport (succinate–ubiquinone reductase, Burns et al., 2015; IRAC, 2025). Originally developed as a fungicide by Bayer CropScience, fluopyram was later developed for its nematicidal properties, with a patent that included use against other plant-parasitic nematodes (PPNs) in the family Anguinidae (Hungenberg et al., 2013). Fluopyram is considered a next-generation nematicide, an informal classification of several nematicides that alludes to their potential to help fill the critical need for effective nematode management tools left by the ongoing phaseout of methyl bromide and other highly toxic, broad-spectrum chemistries that include carbamate and organophosphate nematicides. Compared to older generation nematicides, fluopyram generally exhibits low toxicity to vertebrates and invertebrates due to selective inhibition of succinate dehydrogenase (SDH) function in target pests (Oka, 2020). Several studies have documented a range in nematicidal selectivity and efficacy across evaluated species to fluopyram, including both paralytic effects on mobile life stages and ovicidal hatching inhibition (Heiken, 2017; Schleker et al., 2022). While fluopyram may negatively affect non-target nematodes when applied to diverse communities (e.g., soil-dwelling nematodes in turfgrass systems; Waldo et al., 2019), Lcm remains the only nematode species known to specialize on beech leaves and buds.

In a previously published 2021–2022 field trial conducted at an Ohio nursery, infested European beech trees (*Fagus sylvatica* L.) treated with fluopyram-based foliar applications showed a reduction in BLD symptomatic canopy from an average of 48% to 10%, while another experimental treatment (abamectin + horticultural oil) and non-treated controls exhibited average increases in BLD symptoms of 22% and 16%, respectively. These results were consistent with nematode counts from dormant buds of the same trees, showing that fluopyram-treated trees had roughly 95%–96% fewer Lcm per gram of bud tissue than other treatments. While this initial field trial used the turfgrass nematicide indemnify (34.5% fluopyram; Bayer Environmental Science, Cary, NC) at a rate of 0.7 mL/L (8.5 fl. oz./100 gal.), laboratory assays also confirmed efficacy using the fungicide product Broadform (21.4% fluopyram, 21.4% trifloxystrobin; ENVU, Cary, NC) applied at 0.6 mL/L (8 fl. oz./100 gal.), achieving a 99% reduction in Lcm relative to non-treated controls (Loyd et al., 2024). These initial field and lab studies demonstrated that fluopyram can be used to effectively target Lcm in beech foliage and served as an encouraging first step, but necessitate the need for further research to refine and develop an informed management program. Several key questions emerged during the initial years of exploring chemical management of BLD using fluopyram, which are the focus of the experiments described herein.

First, will foliar fluopyram applications be consistently effective across a range of beech growing environments? Beech trees affected by BLD occur across a wide geographic range and include various age and size classes in nurseries, landscapes, and forests. These variables may introduce unexpected management constraints, especially where accessibility, cost, and labor are limiting factors. Second, what is the optimal application dose that balances efficacy, cost, and environmental stewardship? In vitro dose-response assays demonstrated a high sensitivity of fluopyram to Lcm (EC_50_ = 1.2 ppm), suggesting that significantly lower field rates may be effective (Loyd et al., 2024). Field validation is needed to instigate necessary label amendments. Third, are there alternative application timings or a reduced number of applications that offer equal or improved efficacy? While current fluopyram application programs aim to target mobile stages of Lcm in mid-to-late summer foliage, there may be benefits from early-season applications. For example, fluopyram may possess egg hatch inhibition activity against Lcm, as it does for some other plant-parasitic nematode species, which could serve to suppress Lcm populations prior to peak development and reduce the number of necessary applications, as well as the likelihood of missing the target window prior to Lcm migration events to buds.

Given the variability in beech trees and growing situations affected by BLD, integrating chemical management tools will require long-term research to optimize programs and provide support for appropriate product labeling. This study guides contexts where foliar applications of a fluopyram-based nematicide may be suitable as a practical part of a BLD integrated pest management (IPM) program such as in managed landscapes, nurseries, and ex situ conservation in arboreta.

## Materials and Methods

### Experiment 1: Summer fluopyram program, 2022–2023

Three mixed hardwood forests on private land were selected for this study, each containing minimally disturbed beech groves affected by BLD and exhibiting no other primary health concerns. A total of 68 *F. grandifolia* trees were included across all three sites, located in Chagrin Falls, OH (n = 21), Fairfield, CT (n = 19), and New London, CT (n = 23). Trees were inventoried using the data collection platform Fulcrum (https://www.fulcrumapp.com/) on 19 July 2022 (Chagrin Falls, OH), 21 July 2022 (Fairfield, CT), and 20 July 2022 (New London, CT). Diameter at breast height (DBH) in centimeter, percentage of canopy exhibiting BLD symptoms (primarily banding and severe stunting), percentage of canopy exhibiting fine twig dieback, and a georeferenced point were recorded during these inventories. Ratings were based on a consensus visual rating between three or more certified arborists familiar with BLD symptoms (foliar and fine twig dieback) and expressed as a percentage affected (0%–100%) in 5% increments, rounded up to a minimum of 5% if any BLD symptoms were observed. These variables have been used in other BLD management field trials (Loyd et al., 2025), providing consistency and ease of comparison.

The Chagrin Falls, OH, and Fairfield, CT, sites were managed forest settings with low to moderate beech density, whereas the trees in New London, CT, were in a highly dense, beech-dominated forest. Understory or small- to medium-sized beeches were selected in most cases to allow for full coverage from the foliar applications. Trees were on average 13.9 cm (SE = 1.1), 5.2 cm (SE = 0.8), and 13.6 cm (SE = 3.8) DBH at Chagrin Falls, OH, Fairfield, CT, and New London, CT, respectively. At each location, trees were randomly assigned to one of two treatment groups: fluopyram treatment (Summer Program) or a non-treated control group.

Three foliar applications of fluopyram and one application of foliar phosphite were applied to the selected treatment trees starting just prior to the anticipated dispersal period of Lcm, when they begin moving from the leaf bands to the buds (i.e. mid-summer; Reed et al., 2020). All treatments were full-cover canopy sprays applied to runoff, using a hydraulic sprayer or battery-powered backpack sprayer appropriate for the tree size and site access. Fluopyram was applied using the product Broadform (ENVU), containing 21.4% fluopyram and 21.4% trifloxystrobin, at a rate of 0.63 mL/L (8 fl. oz./100 gal.). The program consisted of four foliar applications approximately 21 d apart, with the first, third, and fourth applications made with Broadform. All Broadform applications included a spreader-sticker (Lesco Spreader-Sticker; Lesco, Inc., Cleveland, OH) at a rate of 0.5 mL/L (6 fl. oz./100 gal.). A foliar application of Reliant (Quest Products Corp., Linwood, KS) at a rate of 5 mL/L (2 qt./100 gal.) served as a rotation product between the first and second Broadform applications. This rotation was included to comply with the resistance management guidelines of the Section 3, EPA-approved Broadform label. Treatment application dates for the 2022 season were as follows: Chagrin Falls, OH: 26 July, 16 August, 6 September, and 27 September; Fairfield, CT: 27 July, 17 August, 7 September, and 28 September; New London, CT: 28 July, 18 August, 8 September, and 3 October.

To quantify the amount of Lcm present in the overwintering buds, twig samples were collected in mid-March to early April 2023 and shipped overnight to the Bartlett Tree Research Laboratories in Charlotte, NC. Samples from each tree consisted of 10–12 live, 15–20 cm long terminal twig sections bearing buds and were stored in a 4°C refrigerator for <1 week prior to processing. For each sample, six buds were removed from randomly selected twigs, weighed, and dissected using forceps. Opened buds were submerged in 10 mL of distilled water in 60 mm diameter Petri dishes and held in the dark for 24 hr. Nematodes were counted under a dissecting microscope with a light mounted below the Petri dish. Counts were standardized as Lcm/g of fresh bud tissue. After one growing season post-treatment, the new flush of foliage was again visually evaluated for disease severity on 14 June 2023 (Fairfield and New London, CT) and 2 August 2023 (Chagrin Falls, OH).

Percent canopy with BLD symptoms and fine twig dieback were square root transformed to normalize variance prior to conducting analysis of variance (ANOVA) for treatment effects. Analyses were conducted independently for each site and year due to a significant location effect. Dormant bud counts were transformed by log (x + 1) to normalize variance before conducting an ANOVA to determine if there was a treatment effect at each site independently. For significant ANOVAs, means were separated with Student’s *t*-test means separation. All analyses were performed using JMP 17.2 (JMP Statistical Discovery LLC, Cary, NC).

### Experiment 2: Fluopyram application rate study, 2023–2024

In the summer of 2023, a field experiment was established to evaluate fluopyram dosage rates, using a similar foliar program timing as described in Experiment 1, consisting of four late-season applications targeting the Lcm dispersal period. For this experiment, American and European beeches were provided by a private nursery located in Riverhead, Long Island, NY. The rows of beech had been field-grown under standard nursery management practices, including overhead irrigation as needed, periodic fertilizer applications, and shaping to maintain form. The trees were approximately 10–15 years old. BLD was well-established in the area and had been present since symptoms were first observed during the 2019 season (Volk and Martin, 2023).

Prior to assigning treatments, disease severity was assessed by estimating the percent canopy with BLD symptoms and fine twig dieback. As previously described in Experiment 1, visual ratings were based on a consensus among at least three certified arborists familiar with scouting for BLD. Only trees with BLD foliar symptoms and no significant confounding problems (e.g., beech bark disease, *Phytophthora* canker, etc.) were selected. Of the 50 beech trees selected, 42 were *F. sylvatica* and 8 were *F. grandifolia.* The *F. sylvatica* selections were identified as *sensu stricto* (*n* = 3), “Dawyck Purple” (*n* = 30), and “Dawyck Green” (*n* = 9). Because trees were planted in rows on approximately 3–4 m centers, non-included trees were left as buffers to avoid interplot interference from potential pesticide drift. This resulted in 10 replicates across five treatments distributed as a randomized complete block design, including numerous buffer trees.

Treatments consisted of four rates of fluopyram applied as Broadform and a non-treated control group. Broadform treatment rates were 0.47 mL/L, 0.32 mL/L, 0.16 mL/L, and 0.08 mL/L (equivalent to 6 fl. oz./100 gal, 4 fl. oz./100 gal, 2 fl. oz./100 gal, and 1 fl. oz./100 gal). These rates represent a 25%, 50%, 75%, and 87.5% dose reduction, respectively, compared to the label rate of 0.63 mL/L (8 fl. oz./100 gal.). In accordance with the 2(ee) recommendations, all Broadform applications included a spreader-sticker (Lesco Spreader-Sticker; Lesco, Inc.) at a rate of 0.5 mL/L (6 fl oz./100 gal). Treatments were randomly assigned within each group of five trees along the rows. The Broadform foliar program was composed of four total applications starting in late July of the 2023 field season. Broadform application dates were made on 27 July, 6–7 September, and 27–28 September. A foliar phosphite application using Reliant, applied at 5 mL/L on 16–17 August, served as a rotation product between the first and second Broadform applications. All foliar applications were made with a gas or battery-powered backpack sprayer, applied from three points around each tree to ensure consistent coverage of the entire canopy, and applied to the point of runoff. Record-keeping by the applicator included weather conditions and product volume used for each treatment at each application date.

On 27 September 2023, just prior to the final foliar application, one twig (15–20 cm long) from each cardinal direction was collected from each tree for nematode quantification from leaves and maturing buds. Samples were packed in a cooler and sent overnight to the Bartlett Tree Research Laboratories in Charlotte, NC, where they were stored at 4°C refrigerator until processing, which was completed within a week of arrival. Five leaves with heavy BLD banding symptoms were selected from each sample, and a standard 6 mm hole punch was used to excise 10 pieces from the banded leaf tissue distributed across the 5 leaves. These leaf pieces were floated in 10 mL of distilled water in a 60 mm Petri dish and held in the dark for 24 hr prior to counting. Lcm were counted under a dissecting microscope with a light mounted below the Petri dish. Counts were standardized by surface area of the 10 leaf punches (2.8 cm^2^) as Lcm/cm^2^.

Following this leaf sample processing, the sample material, four twigs from each tree, was sent to the Mycology and Nematology Genetic Diversity and Biology Laborator y (USDA-ARS, Beltsville, MD) for further evaluation of Lcm populations and damage occurring to buds. Five trees from each fluopyram dosage group and eight non-treated control trees were selected randomly for this work. At least 10 individual buds (10–13, average: 10.1) were randomly collected from the twigs of each sample, then processed and qualitatively rated for damage associated with Lcm, as described in Wolf and Vieira (2024). For each dissected bud, every bud scale was classified using a rating system of 0–4, where 0 is asymptomatic bud scales and 4 is highly symptomatic bud scales, and 1–3 is intermediate ratings. Eggs and vermiform Lcm were quantified per gram of bud tissue. For buds with <100 nematodes, all nematodes within each bud were counted. For buds with >100 nematodes, extracted nematodes were first diluted in 2 mL of water, then three aliquots of 30 µL were used to count nematodes, before extrapolating back to the number of nematodes/2 mL.

In the season after treatment, on 11 June 2024, trees were re-evaluated for percent canopy with BLD symptoms and percent canopy with fine twig dieback to determine if the 2023 season treatments effectively reduced damage to the subsequent flush of developing foliage.

Pre- and post-treatment percent canopy with BLD symptoms and percent canopy with fine twig dieback were analyzed using an ANOVA for treatment effect across the two disease severity measurements for each time point independently. Bud scale ratings (0–4) were analyzed across treatment groups using a non-parametric Wilcoxon test. The total number of Lcm in BLD banded leaf tissue was square root transformed to normalize variance prior to conducting a one-way ANOVA across the treatment groups. Similarly, total Lcm as eggs or vermiform stage from maturing buds was square root transformed to normalize variance before conducting an ANOVA to determine if there was a treatment effect for eggs and vermiform nematodes independently. For significant ANOVAs, means were separated with Tukey’s HSD mean separation. For the non-parametric analysis of bud scale damage, means were separated with a Wilcoxon pairwise analysis. All analyses were performed using JMP 17.2 (JMP Statistical Discovery LLC, Cary, NC).

### Experiment 3: Fluopyram seasonal timing study, 2023–2024

In summer 2023, two sites with beech trees affected by BLD and no other primary health concerns were chosen to evaluate different foliar application timings using Broadform as a fluopyram-based product. One site was a privately owned, field-grown nursery in Perry, OH, where beech trees approximately 10–15 years old were grown in rows. Here, 24 beeches were selected from a variety of *F. sylvatica* cultivars, including “Dawyck Purple,” “Purple Fountain,” “Copper,” and “Red Obelisk.” Another site was a private woodland in Chardon, OH, where *F. grandifolia* was a dominant component of a mixed hardwood natural forest. Here, 32 understory beeches were selected, for a total of 56 trees included in the study. On 15 May 2023 (Perry, OH) and 16 May 2023 (Chardon, OH), trees were inventoried and assessed for percent canopy with BLD symptoms. Initial percent fine twig dieback was assessed only at the Chardon, OH site. At each location, the experiment was set up as a randomized complete block design, with one of four treatments randomly assigned to groups of four trees within close proximity. Thus, six blocks comprised of *F. sylvatica* were located at the Perry, OH site, and eight blocks of *F. grandifolia* at the Chardon, OH site. The four treatments, designed to target different initiation timings of a Broadform program during the growing season, were as follows: Spring (*n* = 14), Spring and Summer (*n* = 15), Summer (*n* = 13) and a non-treated control group (*n* = 14).

The programs consisted of two Broadform applications applied at a rate of 0.63 mL/L (8 fl. oz./100 gal.), with a foliar phosphite application (Reliant, applied at 5 mL/L) between the two Broadform applications. In accordance with 2(ee) recommendations, Broadform applications included a spreader-sticker (Lesco Spreader-Sticker; Lesco, Inc.) at a rate of 0.5 mL/L (6 fl. oz./100 gal.). Non-treated controls received no applications. All treatments were made to the entire canopy (tree height ranged from approximately 3–6 m) using a battery-powered backpack sprayer until the point of runoff. All treatments occurred during the 2023 growing season.

The Spring program application dates were as follows: 23 May, 21 June, and 15 July for Chardon, OH; 2 June, 21 June, and 15 July for Perry, OH. The Spring and Summer program applications were made at both sites on 29 May, 21 June, and 26 July. Finally, for the Summer program, applications were made at both sites on 26 July, 17 August, and 8 September. Following the completion of the three programs, samples were collected on 29 September 2023 to quantify the amount of Lcm present in the symptomatic tissue, following the methods outlined in Experiment 2. From each tree at both sites, 10–12 live, 15–20 cm long twigs exhibiting BLD leaf banding were collected and shipped overnight to the Bartlett Tree Research Laboratories in Charlotte, NC. Prior to processing, the twig samples were stored in a 4°C refrigerator for <1 week. From each sample, five leaves with heavy banding symptoms were selected, and a standard 6 mm hole punch was used to excise 10 pieces from the banded leaf tissue. These leaf pieces were floated in 10 mL of distilled water in a 60 mm Petri dish and held in the dark for 24 hr prior to counting. Lcm were counted under a dissecting microscope with a light mounted below the Petri dish. Counts were standardized by surface area of the 10 leaf punches (2.8 cm^2^) as Lcm/cm^2^. Similarly, in early spring 2024 (28 March for Chardon, OH, and 29 March for Perry, OH), 15–20 cm long twig samples were again collected from all trees to quantify the amount of Lcm in late dormant season bud tissue. For each sample, six buds were removed from randomly selected twigs, weighed, and dissected with forceps to open bud sheaths. Opened buds were submerged in 10 mL of distilled water in 60 mm diameter Petri dishes and held in the dark for 24 hr. Nematodes were counted under a dissecting microscope with a light mounted beneath the dish. Counts were standardized by mass of fresh bud tissue as Lcm/g.

After one growing season post-treatment, trees at both locations were visually evaluated for disease severity, including percent canopy with BLD symptoms and percent canopy with fine twig dieback. Ratings were based on a consensus visual estimate amongst three or more certified arborists familiar with BLD scouting, as previously described. Pre- and post-treatment percent canopy with BLD symptoms (Perry and Chardon, OH) and percent canopy with fine twig dieback (Chardon, OH site only) were analyzed using a restricted maximum likelihood (REML) mixed model across treatment program timings independently with site location as a random effect and treatment program timing as a fixed effect in a general linear mixed model. Lcm/cm^2^ from banded leaf tissue was square root transformed to normalize variance prior to conducting a REML mixed model analysis, with treatment program timing as a fixed effect and location as a random effect. Similarly, dormant bud counts were square root transformed and analyzed using a REML mixed model with the same fixed and random effects. For significant fixed effects in mixed models, means were separated using Tukey’s HSD. All analyses were performed using JMP 17.2.

## Results

### Experiment 1: Summer and fall fluopyram program, 2022–2023

During the 2022 season, pre-treatment BLD symptom ratings were similar across all three sites for percent canopy with foliar BLD symptoms (*P* = 0.9895) and percent canopy with fine twig dieback (*P* = 0.8362), and within each site across treatment groups. Dormant bud nematode counts, which are used to assess treatment efficacy that help to predict next-season visual symptom severity, showed a significant reduction in Lcm populations of treated trees at all sites. At Chagrin Falls, OH, and Fairfield, CT, average Lcm counts from buds of treated trees were <1% of those in neighboring non-treated trees, while a 39% reduction was observed at the New London, CT site. However, overall Lcm bud counts at the New London, CT site, were extremely high, suggesting that despite a significant treatment effect, Lcm levels remained sufficient to drive symptom development and continued disease progression (Table 1). This aligns with previous findings examining the correlation between nematode numbers in dormant buds and the resulting severity of bud damage (Vieira et al., 2023).

**Table 1:** Results of multi-site summer fluopyram program study, 2022–2023.

**Location**	**Treatment**	**Dormant bud counts (Lcm/g)^a^**	***P*-value^b^**
Chagrin Falls, OH	Non-treated	837.56 ± 331.01 A	<0.0001
	Summer Program	7.62 ± 5.36 B	
Fairfield, CT	Non-treated	2590.49 ± 1258.51 A	=0.0005
	Summer Program	3.78 ± 1.52 B	
New London, CT	Non-treated	4861.3 ± 1384.56 A	=0.0017
	Summer Program	1909.26 ± 1404.84 B	

Direct impact on *Litylenchus crenatae mccannii* (Lcm) populations was measured using nematode extractions from dormant buds in late winter following summer treatments. Counts were standardized by bud mass of the sample.

a Raw means plus/minus standard error are shown. Means followed by the same letter are not significantly different by Student’s *t*-test, *P* < 0.05, using transformation by log (x + 1) prior to analysis.

b Where the overall ANOVA across all sites showed variance, Prob > *F* is shown for each location using Student’s *t*-test.

ANOVA, analysis of variance.

In the season following treatment, BLD symptom ratings differed significantly among sites for both percent canopy with foliar BLD symptoms (*P* < 0.0001) and percent canopy with fine twig dieback (*P* = 0.008). At both Chagrin Falls, OH, and Fairfield, CT sites, treated trees exhibited markedly reduced BLD symptoms and fine twig dieback compared to non-treated controls, which showed worsening disease progression (Table 2). In contrast, trees at the New London, CT site, showed a dramatic increase in both symptom severity and dieback across both groups, consistent with the overall high Lcm levels observed in the early spring bud samples.

**Table 2:** Results of multi-site summer fluopyram program study, 2022–2023.

**Location**	**Treatment**	**% Canopy BLD symptoms**					**% Fine twig dieback**				
	
		***N***	**2022^a^**	***N***	**2023**	***P-value* ^b^**	***N***	**2022**	***N***	**2023**	***P-value***
Chagrin Falls, OH	Non-treated	9	64.44 ± 7.66 A	9	88.89 ± 3.51 A	= 0.0005	9	40.56 ± 6.69 A	9	51.67 ± 5.59 A	= 0.0143
	Summer Program	12	69.17 ± 5.11 A	11	34.18 ± 10.35 B		12	47.5 ± 6.17 A	11	28.64 ± 6.47 B	

Fairfield, CT	Non-treated	8	75.63 ± 8.21 A	4	84 ± 10.02 A	< 0.0001	8	63.75 ± 5.96 A	4	77.00 ± 2.38 A	= 0.0046
	Summer Program	11	66.36 ± 8.66 A	11	5.91 ± 0.61 B		11	56.82 ± 5.81 A	11	27.73 ± 7.53 B	

New London, CT	Non-treated	9	8.33 ± 1.44 A	8	76.06 ± 9.27 A	= 0.9009	9	5 ± 0 A	8	50.31 ± 11.11 A	= 0.8592
	Summer Program	14	13.93 ± 3.28 A	14	75.75 ± 4.49 A		14	5 ± 0 A	14	51.07 ± 6.86 A	

Summer program efficacy on BLD management was measured by the percent canopy with foliar BLD symptoms and the percent canopy with fine twig dieback or thinning, as determined by visual rating consensus in the season of treatment and following treatment.

a Raw means plus/minus standard error are shown. Means followed by the same letter are not significantly different by Student’s *t*-test, *P* < 0.05, using square root transformation prior to analysis.

b Where the overall ANOVA across all sites showed variance with the season (2023 for both assessments), Prob > *F* is shown for each location using Student’s *t*-test.

ANOVA, analysis of variance; BLD, beech leaf disease.

### Experiment 2: Fluopyram application rate study, 2023–2024

**Figure 1:**
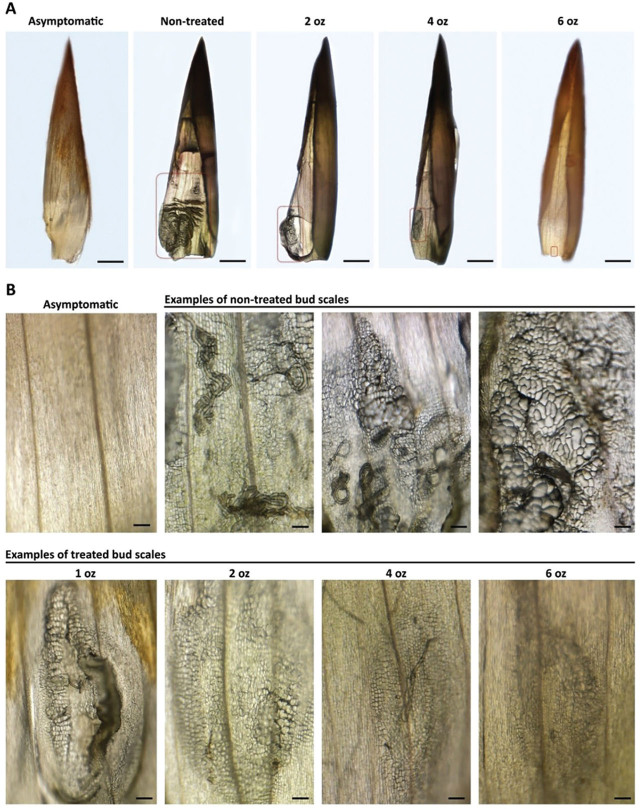
Bud scale morphology of asymptomatic, non-treated, and fluopyram-treated BLD buds of European beech (*Fagus sylvatica*). (A) Stereoscope images of asymptomatic, non-treated, and treated individual bud scales (i.e., number of ounces/100 gallons) infected with *Litylenchus crenatae mccannii* (Lcm) nematodes showing different levels of cell damage. The red rectangle indicates the bud scale damage area in each treatment. (B) Stereoscope images showing enlarged bud scale cells associated with the presence of nematodes on non-treated bud scales versus bud scale enlargement associated with the different fluopyram treatments applied in this study. Scale bars: (A) 1 mm; (B) 100 µm. Treatments in ounces shown as standard tank-mix rates of 1 fl. oz./100 gal., 2 fl. oz./100 gal., 4 fl. oz./100 gal., and 6 fl. oz./100 gal., equivalent to 0.08 mL/L, 0.16 mL/L, 0.32 mL/L, and 0.47 mL/L, respectively. BLD, beech leaf disease.

In the fluopyram application rate study, all four treatments at doses below the label rate of 0.63 mL/L (8 fl. oz./100 gal.) performed equally well in reducing Lcm populations in the treated foliage, with a >99% reduction observed after two of three applications. Counts of active Lcm and eggs from bud samples collected in late summer showed a >98% reduction across all treatments, with a trend toward lower numbers at each consecutively higher dose. Corresponding bud damage ratings were also significantly reduced compared to the non-treated control, although none of the treatments eliminated the risk of Lcm ingress into buds and subsequent damage (Table 3). The significantly reduced number of nematodes recovered from the subset of dissected buds indicated a markedly lower degree of damage to the bud scales in the different treatments in comparison to the non-treated control buds (Fig. 1A). This was further supported by the limited number of bud scales showing cellular abnormalities, such as the characteristic enlarged cells typically observed following the interaction between Lcm and bud scale tissues (Fig. 1B).

**Table 3:** Results of fluopyram application rate study performed on Long Island, NY, 2023–2024.

**Treatment**	***N***	**Lcm/cm^2^**	***N***	**Lcm/g**	**Lcm eggs/g**	**Damage rating^b^**
		**2023––Foliage^a^**		**2024––Bud**	**2024––Bud**	**2024––Bud scale**
Broadform (0.08 mL/L)	10	0.3 ± 0.3 B	5	26.13 ± 6.95 B	36.91 ± 16.27 B	0.61 ± 0.11 B
Broadform (0.16 mL/L)	10	0 ± 0 B	5	13.25 ± 6.33 B	51.32 ± 36.57 B	0.51 ± 0.14 B
Broadform (0.32 mL/L)	10	0 ± 0 B	5	11.7 ± 5.86 B	4.17 ± 4.17 B	0.35 ± 0.13 B
Broadform (0.47 mL/L)	10	0 ± 0 B	5	2.15 ± 1.11 B	0.76 ± 0.76 B	0.12 ± 0.05 B
Non-treated	10	95.5 ± 32.04 A	8	8323.4 ± 2531.01 A	4237.17 ± 1771.21 A	1.63 ± 0.21 A
*P*-value^c^		*P* < 0.0001		*P* < 0.0001	*P* = 0.0038	*P* < 0.0001

Direct impact on *Litylenchus crenatae mccannii* (Lcm) populations was measured using live Lcm extraction from foliage following two of three treatments, Lcm (active and eggs) extracted from late summer developing buds, and bud scale damage ratings. Count data were standardized by foliage area and bud mass of material in each sample. Product applied as Broadform using four application rates lower than the highest label rate of 0.63 mL/L (8 fl. oz./100 gal.). Application rates were 0.08 mL/L, 0.16 mL/L, 0.32 mL/L, and 0.47 mL/L, equivalent to standard tank-mix rates of 1 fl. oz./100 gal., 2 fl. oz./100 gal., 4 fl. oz./100 gal., and 6 fl. oz./100 gal., respectively.

a Raw means plus/minus standard error are shown. Means followed by the same letter are not significantly different, Tukey HSD, *P* < 0.05, using square root transformation for all count data prior to one-way ANOVA and means separation. For the non-parametric analysis of bud scale damage, means were separated with a Wilcoxon pairwise analysis.

b Damage ratings shown as treatment means using the methodology described in Wolf and Vieira (2024).

c *P*-value is based on a one-way ANOVA of treatment groups.

ANOVA, analysis of variance.

**Figure 2:**
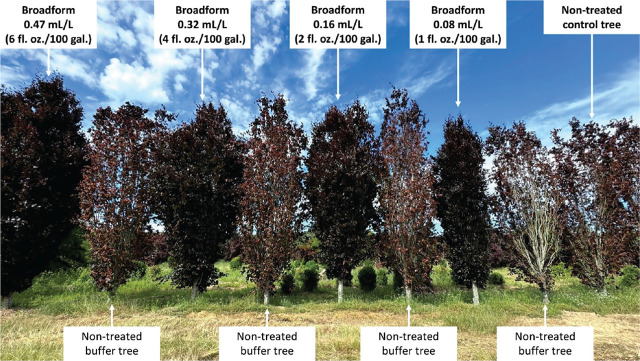
At a private nursery on Long Island, NY, several rows of fastigiate European beech (*Fagus sylvatica* cvs) were used for the fluopyram foliar application rate study. These nine beeches illustrate one treatment block from the study, using the product Broadform to treat BLD caused by the foliar nematode *Litylenchus crenatae mccannii* (Lcm). From left to right, the treatment groups and Broadform application rates (including standard tank-mix rates) were as follows: 0.47 mL/L, non-treated buffer; 0.32 mL/L, non-treated buffer; 0.16 mL/L, non-treated buffer; 0.08 mL/L, non-treated buffer; and non-treated control. Image taken 11 June 2024. BLD, beech leaf disease.

In the season following treatment, disease ratings showed similar reductions in symptom severity across all treatment groups compared to the non-treated control. Treatment effects were consistent across both *F. grandifolia* and *F. sylvatica*, with species remaining a non-significant model effect in both pre-treatment ratings and post-treatment analysis. Treated beech, which averaged 81.9% canopy with BLD symptoms in the pre-treatment ratings, fell to an average of 9.3% post-treatment, representing a nearly 89% decrease in foliar symptoms. Treated beech also exhibited less dieback and had visibly fuller canopies. In contrast, non-treated beech continued to decline, with increased canopy symptoms and fine twig dieback (Table 4). Stark differences between treated and untreated trees, including both the control group and buffer trees, were evident. Treated trees retained their full spring flush of growth, while all untreated trees showed significant canopy thinning and were producing a weak secondary flush of growth (Fig. 2).

**Table 4:** Results of fluopyram application rate study performed on Long Island, NY, 2023–2024.

**Treatment**	***N***	**% Canopy BLD symptoms**		**% Fine twig dieback**	
	
		**2023^a^**	**2024**	**2023**	**2024**
Broadform (0.08 mL/L)	10	77.1 ± 7.15 A	9.3 ± 2.6 B	4.8 ± 2.92 A	1.3 ± 0.68 B
Broadform (0.16 mL/L)	10	83.1 ± 6.36 A	10.9 ± 2.39 B	6.4 ± 2.54 A	0.8 ± 0.55 B
Broadform (0.32 mL/L)	10	83 ± 6.08 A	10.23 ± 2.62 B	7.9 ± 3.05 A	1.8 ± 0.76 B
Broadform (0.47 mL/L)	10	84.4 ± 5.96 A	6.6 ± 1.49 B	5 ± 1.95 A	2.5 ± 2.01 B
Non-treated	10	84.38 ± 7.24 A	89.3 ± 2.99 A	6.5 ± 1.92 A	22.4 ± 7.01 A
*P*-value^b^		*P* = 0.9300	*P* < 0.0001	*P* = 0.9071	*P* < 0.0001

Treatment efficacy on disease management was measured by the percent canopy with foliar BLD symptoms and the percent canopy with fine twig dieback or thinning, as determined by visual rating consensus in the season of treatment and following treatment. Product applied as Broadform using four application rates lower than the highest label rate of 0.63 mL/L (8 fl. oz./100 gal.). Application rates were 0.08 mL/L, 0.16 mL/L, 0.32 mL/L, and 0.47 mL/L, equivalent to standard tank-mix rates of 1 fl. oz./100 gal., 2 fl. oz./100 gal., 4 fl. oz./100 gal., and 6 fl. oz./100 gal., respectively.

a Raw means plus/minus standard error are shown. Letters following each rating represent levels that are statistically different based on Tukey’s HSD, *P* < 0.05, using square root transformation prior to post hoc means separation.

b *P*-value is based on a one-way ANOVA of treatment groups for each season.

ANOVA, analysis of variance; BLD, beech leaf disease.

### Experiment 3: Fluopyram seasonal timing study, 2023–2024

In the seasonal timing study, foliar sampling in late summer (September 2023) following completion of all management programs revealed a marked decrease in Lcm numbers in treated symptomatic leaf tissue. The Spring and Spring & Summer programs each showed a >99% reduction of Lcm in foliage (Table 5). The Summer program yielded statistically intermediate Lcm counts from leaf tissue, though it still demonstrated a biologically meaningful 79% average reduction compared to the non-treated control. Dormant bud sampling in the following March showed similar trends, with all treatment groups exhibiting substantially less Lcm activity than the non-treated control trees. As in the previous experiments, these results illustrate that while complete eradication was not achieved, all program application timings effectively reduced Lcm populations in the foliage, resulting in less activity in the buds, and drastically affected disease progression.

**Table 5:** Results of fluopyram-based management programs, implemented at three seasonal timings, in Perry, OH, and Chardon, OH, 2023–2024.

**Treatment^a^**	***N***	**Lcm/cm^2^**	**Lcm/g^2^**
	
		**2023––Foliage^b^**	**2024––Bud**
Spring	14	0.25 ± 0.25 B	31.29 ± 17.15 B
Spring & Summer	15	0 ± 0 B	25.73 ± 9.04 B
Summer	13	9.65 ± 3.83 AB	30.46 ± 18.29 B
Non-treated	14	46.24 ± 24.67 A	143.86 ± 54.07 A
*P*-value^c^		*P* = 0.0005	*P* = 0.0105

To measure post-treatment impact on *Litylenchus crenatae mccannii* (Lcm) populations, live Lcm nematodes were extracted from foliage taken in late summer and from late dormant season buds. Count data were standardized by foliage area and bud mass of material in each sample.

a Refer to Materials and Methods in Experiment 3 for exact treatment dates.

b Means plus/minus standard error of the mean are shown. Letters following each rating represent levels that are statistically different based on Tukey’s HSD, *P* < 0.05, using square root transformation prior to post hoc means separation.

c *P*-value derived from REML mixed model analysis examining treatment program as a fixed effect. REML, restricted maximum likelihood.

Post-treatment disease severity ratings aligned with expectations drawn from Lcm populations in late dormant season buds. All treatment program timings significantly reduced canopy BLD symptoms relative to the non-treated control group, with improvement ranging from approximately 78% for the Spring program to 89% for the Spring & Summer program. Non-treated trees also showed some year-over-year improvement, but to a much lesser extent. The percentage of fine twig dieback was statistically similar across all groups within each season. However, during the post-treatment season, trees in the treatment groups experienced approximately 65%–78% less dieback, compared to a 41% reduction in the non-treated group (Table 6).

**Table 6:** Results of fluopyram-based management programs implemented at three seasonal timings in Perry, OH, and Chardon, OH, 2023–2024.

**Treatment^a^**	***N***	**% Canopy BLD symptoms**		***N***	**% Fine twig dieback^c^**	
	
		**2023^b^**	**2024**		**2023**	**2024**
Spring	14	75 ± 5.3 A	18.2 ± 6.2 B	8	25.6 ± 5.6 A	8.94 ± 1.37 A
Spring & Summer	15	54.7 ± 7 A	6 ± 1.2 B	8	24.4 ± 6.5 A	5.63 ± 1.03 A
Summer	13	58.5 ± 7.7 A	10.2 ± 3.7 B	8	30.6 ± 5.4 A	6.75 ± 0.98 A
Non-treated	14	54.3 ± 6.3 A	41.5 ± 7.7 A	8	16.9 ± 4.4 A	9.88 ± 1.63 A
*P*-value^d^		*P* = 0.0982	*P* < 0.0001		*P* = 0.3843	*P* = 0.0966

To measure program efficacy on disease management, the percent canopy with foliar BLD symptoms and the percent canopy with fine twig dieback or thinning were determined by visual consensus ratings during the treatment season and the season following treatment.

a Refer to Materials and Methods in Experiment 3 for exact treatment dates.

b Means plus/minus standard error of the mean are shown. Letters following each rating represent levels that are statistically different based on Tukey’s HSD, *P* < 0.05, using square root transformation prior to post hoc means separation.

c Fine twig dieback ratings were not collected at the Perry, OH site, during the 2023 season. Therefore, only ratings from the Chardon, OH, site, are shown for both 2023 and 2024. No significant differences were observed when including post-treatment site ratings from Perry, OH, in the analysis.

d *P*-value derived from REML mixed model analysis examining treatment program for each season. BLD, beech leaf disease; REML, restricted maximum likelihood.

## Discussion

This research provides timely, field-validated information for the management of BLD, a destructive and rapidly spreading tree disease affecting both American and European beeches, the two most widely cultivated *Fagus* species in North America. The management strategy and nematicidal treatment described herein are intended for arborists, nurserymen, and plant health care specialists working in regions where beeches are grown and maintained. Others who may benefit include conservationists managing native beech populations, curators of living plant collections at arboreta, and information-driven homeowners who grow beech as high-value shade trees, hedges, or in woodlots.

Our initial research (Loyd et al., 2024) suggested that fluopyram, a fungicide that is currently registered as various products for use on ornamental landscapes, nurseries, and beech nut plantings as a fungicide, could also be employed for its well-studied nematicidal properties against the causal agent of BLD. These combined field and lab findings supported an application and subsequent approval of a FIFRA Section 2(ee) recommendation for Broadform® (Bayer, EPA Reg. No. 432-1537, October 2022), as well as a FIFRA Section 24(c) recommendation for Broadform® use in Nassau and Suffolk counties, New York (Bayer, EPA Reg. No. 432-1537, SLN. No. NY-220002, October 2022). Both of these special-use labels “For Control of Beech Leaf Disease on Beech Trees” provided an application rate of 8 fl. oz./100 gal., equivalent to the highest label rate, based on available data at the time. As is the typical best practice when repurposing an existing management tool for a new host or pest, our research focus quickly shifted from proof-of-concept to two primary goals: (1) evaluating efficacy across a range of locations and beech-growing environments, and (2) optimizing treatment programs to reduce product usage and environmental exposure while maintaining efficacy.

The Summer fluopyram program (Experiment 1) was most similar in methodology to the initial fluopyram field trial in Perry, OH (Loyd et al., 2024), employing the highest label rate in a rigorous, four-spray program initiated in late July at 21-d intervals. Two sites with well-established BLD responded strongly, showing a significant reduction in Lcm bud populations and corresponding symptom improvement in the following seasons. However, a third site, which had minimal observable BLD in the season of treatment, experienced a dramatic increase in BLD symptoms across all treated and non-treated control trees. Several factors may explain this disparity in treatment outcome. First, the New London, CT site, appeared to lie along the expanding front of BLD during the study period. Despite treatment-related reductions, Lcm bud counts remained extraordinarily high, and symptom severity increased sharply year-over-year. Notably, during the same season in which we inventoried the site and recorded minimal symptoms prior to making treatment applications, Dr. Robert Marra aptly described the “2022 BLD Hell-scape!” approximately 50 miles to the east, where beech forest sites underwent similarly rapid, single-season changes in BLD severity (Marra, 2023). While this raises interesting questions about Lcm population dynamics at the disease front, we believe a second explanation is more relevant from a management perspective.

All beeches used in the trial at the New London site were relatively small, densely growing understory beeches beneath a canopy of mature, spreading beech. Both overstory and understory trees prior to treatment had minimal BLD symptoms. By post-treatment assessments the following season, the beech in this forest, including the overstory beech, displayed extensive foliar BLD symptoms and canopy thinning. In the context of localized Lcm movement (Goraya et al., 2024), it is likely that treatment effects on understory beech in the study had limited effects on the overstory produced inoculum that moved in rain and wind onto the understory trees in the trial. The residual activity of the fluopyram as a contact nematicide was likely reduced during peak emergence of Lcm, and since fluopyram does not move into bud tissue (Loyd et al., 2024), Lcm from surrounding sources was able to colonize developing buds directly, regardless of foliar treatments made to the understory trees in the study. In comparison to the other two sites in this study, the New London site had a significantly higher beech density, which has been suggested as a major driver of disease progress in forests (Shepherd et al., 2025).

These site-specific outcomes reinforce the need to take into account both relative reduction and absolute Lcm numbers when evaluating treatment efficacy. Biological significance should be assessed not only by percent reduction but also by the resulting bud protection and next-season canopy health. For practical decision-making, it is advisable that fluopyram-based treatments may not be appropriate where Lcm inoculum pressure from adjacent, untreated beech is exceptionally high, as from dense beech stands, which have the potential to overwhelm treatment effects.

The fluopyram application rate study (Experiment 2) yielded strong evidence supporting our goal of reducing fluopyram dose while maintaining efficacy that is similar to the highest label rate (0.64 mL/L, equivalent to 8 fl. oz./100 gal.) used in previous field trials (Experiment 1 and Loyd et al., 2024). Following the completion of Experiment 2, we assisted with the generation of a revised FIFRA Section 2(ee) recommendation for the product Broadform® (ENVU, EPA Reg. No. 101563-158, June 2024). This recommendation increased the application interval to 21 d and permitted a rate reduction of 50% compared to the previously recommended rate. However, the similar reductions in Lcm activity (>98%) and consistency of canopy improvement across all application rates evaluated suggest that lower rates may also be sufficient to maintain beech canopy health and minimize the negative impacts of BLD. By reducing the rate of Broadform and still providing efficacy, pesticide applicators can reduce the environmental impact of intervention as well as the cost of treatment. As is typical of pest management services, the amount of product required per tree is only one of many factors to consider. Total cost may also include the equipment required, service time and labor, applicator technique and certifications, the choice and cost of rotation product, should they be necessary, rates selected, and market prices of all products. It makes sense that these robust data should support lower Broadform rates to manage BLD in beech in the landscape and nurseries.

The results of Experiment 3 demonstrated that our goal of developing effective fluopyram programs for BLD with a reduced number of necessary applications and alternative timings is achievable. While the general application timings of the Summer program (starting in late July) had been explored in previous trials, the Spring program and Spring & Summer program were slightly more effective, although not statistically different than the Summer program when evaluating canopy symptoms. The period of the Lcm life cycle between bud break and late summer is not yet well understood. Observations conducted during early spring indicate that the number of Lcm present in newly expanding symptomatic beech leaves is initially low (Vieira et al., 2023). This early presence coincides with the timing of the nematode’s migration into the developing foliage, suggesting that leaf invasion occurs shortly after bud break. As the season progresses, the sustained interaction between Lcm and the host plant likely contributes to both the intensification of foliar symptoms and the further establishment of nematode populations. Thus, possible targets of late May/early June applications could be direct action against mobile nematode stages, and possibly egg hatch inhibition activity. Future work should investigate the effects of fluopyram on all life stages of Lcm, as these early-season life stages likely represent the lowest point of the population curve and thus the optimal time for control from both efficacy and resistance-avoidance perspectives. The truncated programs in Experiment 3 also demonstrated that significant Lcm reduction and BLD suppression are achievable with only two fluopyram applications during the season, as opposed to three applications used in other trials. Together, the results of Experiments 2 and 3 strongly support a practical and IPM-appropriate shift towards lower-dose fluopyram treatments and timing-optimized applications as a means of responsibly using a highly effective management tool, while reducing product use, costs, and environmental loading.

As BLD management continues to move towards meeting the need for long-term, sustained efficacy, several areas of research should be prioritized. First, efficacy trials should continue to explore novel or untested chemistries, particularly to provide rotation options from the perspective of resistance avoidance. While no pesticide resistance has been documented or observed in Lcm populations or in the context of BLD management, resistance to fluopyram has emerged in other systems following long-term exposure (Kammerer et al., 2023). Second, trials should continue to explore alternative application methods for situations where a foliar application is unsuitable, including tree-injection and soil-injection applications. This need is reflective of the diverse sites and situations where beeches are affected by BLD. Third, wherever BLD eradication is impractical, establishing research-based treatment thresholds is sorely needed to inform the practical goal of suppressing BLD to a level that has minimal long-term consequences on canopy function. Indeed, continued optimization of treatment programs may result in 1–2 nematicide applications on an as-needed basis that adequately suppresses BLD to achieve satisfactory management outcomes.

An additional priority is product development designed for this unusual pest/host system involving woody plants and non-root-infesting nematode pests. Currently, the only fluopyram-containing product registered for BLD management is Broadform, via 2(ee) labeling. This product includes fluopyram as well as the fungicide trifloxystrobin. The latter active ingredient offers no known additional benefit against Lcm. Optimally, plant practitioners would have access to a fluopyram formulation that is labeled as a nematicide for Lcm management and BLD suppression on *Fagus* spp. across a variety of sites that support the use of reduced application rates. Similarly, efficacy and selectivity trials are needed to compare fluopyram with other recently developed nematicides. Of particular interest is cyclobutrifluram, a nematicide inspired by fluopyram that was introduced by Syngenta in 2013 (Jeschke, 2024), but which remains unregistered for uses relevant to the BLD pathosystem. Interestingly, cyclobutrifluram has shown excellent potential as a tree-injection tool to manage pine wood nematode, *Bursaphelenchus xylophilus* (Steiner and Buhrer, 1934), a similarly difficult-to-manage nematode infesting forest and landscape trees (Heydari et al., 2023; Liu et al., 2024). While foliar applications of nematicides such as fluopyram may be appropriate and cost-effective for small to medium-sized beech trees, tree-injection applications generally result in reduced environmental exposure and minimized drift, while maintaining efficacy as has been demonstrated in another developed BLD management tool (Loyd et al., 2025). Cyclobutrifluram shares the mode of action of fluopyram but exhibits relatively higher nematode selectivity, even when compared to the potent nematicides fluopyram, emamectin benzoate, and abamectin in the context of *B. xylophilus* (Liu et al., 2024). This chemistry may therefore offer even further reduced environmental loading while minimizing the destructive impact of BLD.

In summary, this research offers significant improvements for the use of fluopyram as a tool in BLD management. Results confirm that fluopyram is highly effective as a foliar-applied treatment, but successful implementation may depend on local context, including the growing conditions and tree size. Educational opportunities are needed for certified arborists and pesticide applicators who prescribe treatments and decide whether a fluopyram-based foliar program may be appropriate. Additionally, thresholds should be developed to aid in the interpretation of treatment results, prescribing future treatments on an as-needed basis, and meeting appropriate goals for beech tree health. Integrating nematicidal treatments will likely be essential for the immediate need of BLD suppression, but should be considered part of a broader IPM framework that will ultimately include cultural, mechanical, and chemical approaches to management. In a disease system characterized by novel complexities and continued rapid spread in North America, this study brings an improved understanding of site influence and optimized application guidelines, solidifying fluopyram as a valuable nematicidal tool to preserve beech trees affected by BLD.
